# Increased Erythrocytes By-Products of Arginine Catabolism Are Associated with Hyperglycemia and Could Be Involved in the Pathogenesis of Type 2 Diabetes Mellitus

**DOI:** 10.1371/journal.pone.0066823

**Published:** 2013-06-24

**Authors:** Serafín Ramírez-Zamora, Miguel L. Méndez-Rodríguez, Marisela Olguín-Martínez, Lourdes Sánchez-Sevilla, Miguel Quintana-Quintana, Norberto García-García, Rolando Hernández-Muñoz

**Affiliations:** 1 Depto. de Biología Celular, Instituto de Fisiología Celular, Universidad Nacional Autónoma de México (UNAM), Coyoacán, Mexico Distrito Federal, Mexico; 2 Escuela Médico Naval, Secretaría de Marina (Navy), Alvaro Obregón, Mexico Distrito Federal, México; 3 Hospital Naval de Alta Especialidad, Secretaría de Marina (Navy), Alvaro Obregón, Mexico Distrito Federal, México; University of Michigan Medical School, United States of America

## Abstract

Diabetes mellitus (DM) is a worldwide disease characterized by metabolic disturbances, frequently associated with high risk of atherosclerosis and renal and nervous system damage. Here, we assessed whether metabolites reflecting oxidative redox state, arginine and nitric oxide metabolism, are differentially distributed between serum and red blood cells (RBC), and whether significant metabolism of arginine exists in RBC. In 90 patients with type 2 DM without regular treatment for diabetes and 90 healthy controls, paired by age and gender, we measured serum and RBC levels of malondialdehyde (MDA), nitrites, ornithine, citrulline, and urea. In isolated RBC, metabolism of L-[^14^C]-arginine was also determined. In both groups, nitrites were equally distributed in serum and RBC; citrulline predominated in serum, whereas urea, arginine, and ornithine were found mainly in RBC. DM patients showed hyperglycemia and increased blood HbA_1C_, and increased levels of these metabolites, except for arginine, significantly correlating with blood glucose levels. RBC were observed to be capable of catabolizing arginine to ornithine, citrulline and urea, which was increased in RBC from DM patients, and correlated with an increased affinity for arginine in the activities of putative RBC arginase (*Km* = 0.23±0.06 vs. 0.50±0.13 mM, in controls) and nitric oxide synthase (*Km* = 0.28±0.06 vs. 0.43±0.09 mM, in controls). In conclusion, our results suggest that DM alters metabolite distribution between serum and RBC, demonstrating that RBC regulate serum levels of metabolites which affect nitrogen metabolism, not only by transporting them but also by metabolizing amino acids such as arginine. Moreover, we confirmed that urea can be produced also by human RBC besides hepatocytes, being much more evident in RBC from patients with type 2 DM. These events are probably involved in the specific physiopathology of this disease, i.e., endothelial damage and dysfunction.

## Background

Diabetes mellitus (DM) is a worldwide disease frequently associated with high risk of atherosclerosis and renal, nervous system, and ocular damage [1]. Oxidative damage is involved in diabetes and its complications [1]–[3], and reactive oxygen species (ROS) have been implicated in the pathogenesis of DM [4]. Patients with type 2 DM frequently have vascular endothelium dysfunction, associated to hypercholesterolemia, and nitric oxide (NO) deficiency is a major factor contributing to endothelial dysfunction, as has been evidenced in hypertension, tobacco smoking, and malaria [5].

In the same context, increased production of ROS has been related to protein glycation [2] and/or glucose auto-oxidation in DM patients [6]. Glycosylated proteins differ in their biological half-lives and reactivities; serum glycosylated albumin reflects blood glucose levels, since hemoglobin undergoes increased glycation (Hb A_1C_) throughout the life span of red blood cells (RBC), under hyperglycemic conditions [7]. In turn, glycation of proteins can lead to oxidative stress by direct release of superoxide and H_2_O_2_ [8]. Glycated albumin seems to be a more sensitive index of short-term variations of glycemia than Hb A_1C_ during treatment of diabetic patients [9]. High serum malondialdehyde (MDA) and organic hydroxyperoxide concentrations have been observed in patients with ketoacidosis as secondary effects of glycemic disorders [10]. Additionally, increased lipid peroxidation (LP) occurs in membranes of RBC due to an excessive production of ROS and decreased levels of GSH. Hematological alterations in serum and/or blood cells (augmented serum conjugated dienes and lipid peroxides) have been observed in type 2 diabetic patients with vascular complications [11].

It is likely, therefore, that changes in redox state and oxidative stress may have profound effects on blood cells and their function, and RBC are the most abundant and feasible targets for deleterious actions of some metabolites. In this context, growing evidence has shown that physiological levels of NO play an important role in regulating oxidation of metabolic intermediates, insulin sensitivity, and hemodynamics in animals and humans [12]. NO is a key mediator of the immune response [13] and of neurological functions [14]. It is synthesized from L-arginine by tetrahydrobiopterin (BH_4_)-dependent NO synthase [15], and dietary supplementation with L-arginine reduces serum levels of glucose in diabetic rats [16], suggesting that this amino acid and L-citrulline might play roles as novel and potentially effective therapies for obesity, diabetes, and the metabolic syndrome [17]. In fact, arginine administration could be effective in reversing endothelial dysfunction since it has been reported to restore endothelial NO synthesis, decrease superoxide production, reduce vascular oxidative damage, and inhibit platelet aggregation [5], [18].

There are multiple pathways for arginine degradation to produce NO, ornithine, urea, polyamines, proline, glutamate, creatine, and/or agmatine, and these pathways are initiated by arginases, three isoforms of NOS, as well as arginine/glycine amidinotransferase and arginine decarboxylase [15]. Arginine can stimulate insulin release both in vivo and in vitro, suggesting that this amino acid facilitates the action of glucose on insulin secretion [18]. In mammals, the arginase pathway is quantitatively the most important for arginine catabolism; type-I arginase is expressed abundantly in hepatocytes [19] and, to a limited extent, in extra-hepatic cells, including RBC from primates [20]. RBC from healthy subjects can synthesize urea apparently through an arginase-like activity, and the linear rate of urea synthesis along time suggests that extracellular and intracellular arginine equilibrate rapidly in blood cells [21]. Nonetheless, the significance of extra-hepatic urea synthesis is not clear, since RBC contribution to urea synthesis has been estimated to be 1 to 3% of the total urea production.

Arginine-derived NO has been implicated in vascular dysfunction of diabetic patients, in whom this pathological process is characterized by impaired endothelial cell production of the vasodilator and antiplatelet adhesion factor, and/or decreased NO bioavailability [22]. Moreover, increased arginase I activity and expression are associated with diabetes-induced increases in oxidative stress and in initiating feed-forward cycle of diminished NO levels and oxidative stress [23]. Besides, free heme (hemoglobin) impairs L-arginine transport across the RBC membrane and increases its breakdown, contributing to the reduced NO level observed in patients with severe malaria [24].

Based on the aforementioned facts, we hypothesized that fluctuations in serum levels of metabolites are influenced by RBC, and this putative “buffering” property of RBC for removing and/or releasing different metabolites from or into the serum can be altered largely by hyperglycemia and glycosylated by-products, disturbing structure and/or function of RBC.

Therefore, in the present work we tested whether metabolites reflecting oxidative state and NO metabolism are differentially distributed between serum and RBC, and we evaluated also the capacity of isolated RBC to metabolize arginine. For this purpose, we used samples from patients with type 2 DM to determine the impact of this metabolic disease on these parameters.

## Methods

### Patients and Controls

Subjects with type 2 DM, at different stages of the disease, were recruited consecutively from the outpatient clinic at the Naval Medical Center (Ministry of Naval Force). The study group consisted of 90 patients with type 2 (non-insulin-dependent) DM, selected based upon the following: all patients were non-alcoholics, non-smokers, and without regular treatment for diabetes at the start of the study. Patients were apparently free from any renal or liver complications. Ninety age-, gender-, and body weight-matched, non-smoking, non-alcoholic, healthy individuals with no family history of diabetes were studied in parallel as a control group. Following a 12-h overnight fast, all subjects were subjected to blood sampling and clinical assessment by the same investigator (M.L.M.-R.).

### Ethics Statement

This study was carried out in accordance with the Declaration of Helsinki (2000) of the World Medical Association and approved by the Ethics Committee of the Naval Medical Center of the Ministry of Naval Force, after written informed consent was obtained.

### Clinical Tests

In separate blood samples from healthy subjects and diabetic patients, several clinical parameters were quantified: glucose, glycosylated Hb A_1C_, cholesterol, triacylglycerols, and high sensitive C-reactive protein (hs-CRP), as shown in Table 1.

**Table 1:** Clinical parameters of control subjects and patients with type 2 DM.

	Control subjects (n = 90)		Diabetic patients (n = 90)	
	Mean ± SD	Range	Mean ± SD	Range
**Parameter**				
Age (years)	43±12	25–65	49±11	28–70
BMI (kg/m^2^)	25.6±7.2	14.7–37.7	26.4±11.6	13.3–43.7
Glucose (mg %)	83.3±8.1	67.7–107.9	149.5±40.1*	76.6–265.4
Hb A_1C_ (%)	4.6±1.4	2.1–5.8	10.5±1.2*	4.9–14.4
Cholesterol (mg %)	148.4±19.3	96.8–194.5	185.3±25.5*	159.8–263.8
TG (mg %)	142.2±27.2	95.5–186.3	177.1±40.8*	98.1–222.3
hs-CRP (mg/L)	0.30±0.22	0.06–0.69	0.77±0.27*	0.25–1.38

The results are expressed as means ± SD. Abbreviations: BMI, body mass index; TG, triacylglycerols, and hs-CRP, high-sensitive C-reactive protein. Statistics: *p<0.01 as compared to healthy controls.

### Preparation of Acid-extracts from Blood Components

Heparin-anti-coagulated blood was obtained from the experimental groups, and the serum was rapidly separated. Aliquots of serum and RBC package were placed in ice-cold perchloric acid (8% w/v, final concentration). After centrifugation, acid-extracts of serum as well as of RBC were obtained (dilution: 1∶3 v/v blood samples:perchloric acid), and stored at −50°C until use.

### Biochemical Measurements

In neutralized perchloric acid extracts from whole blood, serum, and RBC, thiobarbituric acid reactive substances (TBARS, mainly MDA) were determined according to Hernández-Muñoz et al. [25], citrulline as described by Ceriotti [26], and orrnithine was colorimetrically assayed with the method described by Gaitonde [27]; nitrites were quantified by the Griess reaction [28]. In neutralized perchloric acid-extracts, arginine determination was done by the method described by Gäde [29], and urea was determined according to Kerscher and Ziegenhorn [30]. Total hemoglobin was quantified using the Drabkin’s reagent.

### Preparation of RBC for Incubation Assays

Another set of anti-coagulated blood samples was obtained from healthy subjects (n = 30) and diabetic patients (n = 30) and the serum was rapidly separated and removed after centrifugation at 900 *g* and 4°C for 5 min, the buffy coat was removed, and the erythrocyte pellet was washed four times with two volumes of cold (4°C) buffered solution of 20 mmol/L HEPES (pH 7.42) and containing 0.9% NaCl. Thereafter, RBC were gently resuspended to a 33% hematocrit (Hct) with the same buffered solution and stored at 4°C overnight. RBC were then centrifuged at 900 *g* (4°C) for 5 min and incubated in the same buffered solution (HEPES-NaCl) with the addition of 5 mM glucose and in the presence of L-arginine (0.0 to 0.5 mmol/L) for 60 min at 37°C under continuous gentle shaking (HcT adjusted to 25%). Incubation was stopped placing the 25 mL-Erlenmeyer flasks on ice and RBC were then pelleted by centrifugation at 900 *g* for 10 min. Acid-extracts were performed from aliquots taken from the four washes, the overnight stored solution, as well as from the incubation medium and the total RBC pellet, and all the metabolites described above were measured in them.

### Quantification of (^14^C)-ornithine, (^14^C)-citrulline, and (^14^C)-urea formed from (^14^C)-arginine by Isolated RBC

Aliquots of RBC (Htc of 25%) were incubated in the presence of 0 to 0.5 mmol/L arginine containing 0–2 µCi U-^14^C-arginine (sp. act. 346 mCi/mmol; NEN Radiochemicals, Boston, MA) in buffered NaCl-HEPES solution (pH 7.42) with 5 mmol/L glucose. Incubation at 37°C lasted 60 min and the blood was gently shaken during incubation. At the end of incubation, the whole samples were spun, separated, and acid-extracts were processed as described above. The pH of perchloric acid-extracts was adjusted within 6.0 to 6.5 and free radio-labeled amino acids were identified using non-labeled amino acid carriers, by thin layer chromatography in cellulose plates (Merck de Mexico, D.F.), essentially as described by Kraffcyzk [31]. After revealing the spots corresponding to arginine, ornithine, and citrulline, through a ninhydrin reagent, spots were scrapped from the plate and placed in vials containing tritosol, and then counted for ^14^C dpm in a Packard Tri-Carb Scintillation Spectrometer; results were expressed as nanomols formed after correcting by the calculated specific activity. Neutralized extracts from RBC and supernatants were also incubated in center-well flasks with 1 mg/mL of urease (Sigma Chemical Co., St. Louis, MO) at 37°C for one hour, and released (^14^C)-carbon dioxide was trapped with 10% KOH and counted [21].

### Calculations and Statistics

Concentration of serum and RBC metabolites were calculated as nanomoles or micromoles per milliliter, and expressed as means ± standard deviation (SD). To compare a continuous variable between the two groups, Student’s unpaired t-test and the Mann-Whitney test were used; thereafter, these differences were contrasted with a *t*-test for paired data. Linear regression and correlation coefficients were calculated through a Statistics Program (SigmaStat4), and Pearson correlation analysis was used to test the correlation between various parameters, when indicated.

## Results

### Clinical Parameters

Although no significant difference in body mass index (BMI) was found between both groups, patients with type 2 DM presented fasting hyperglycemia and increased levels of Hb A_1C_ (2 to 3-fold; Table 1). In addition, significant increases in serum levels of cholesterol, triacylglycerols, and hs-CRP were clearly observed in DM patients as compared to healthy subjects (Table 1).

### Metabolites Indicating Oxidative Stress, Generation of NO, and Arginine Catabolism

**Figure 1:**
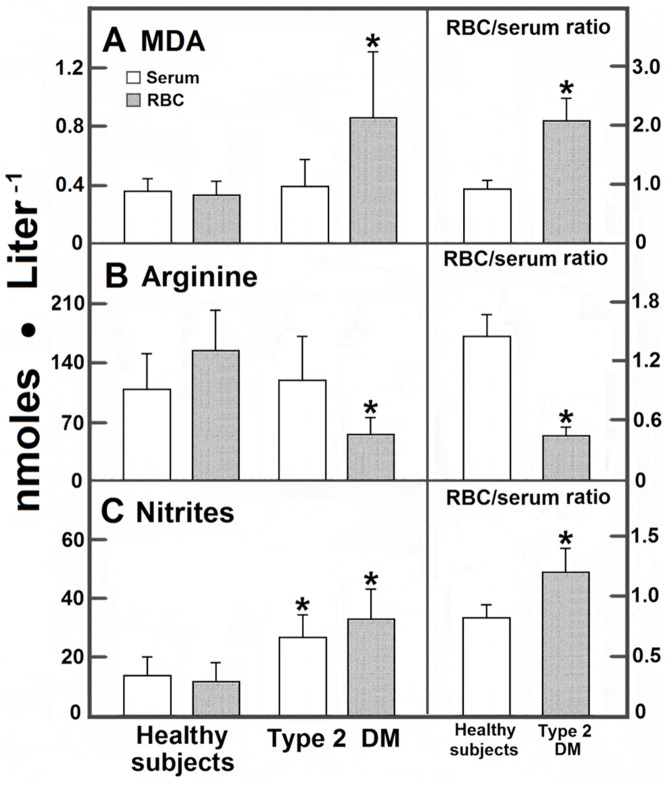
Serum and RBC levels of MDA, arginine, and nitrites from blood obtained from control subjects and patients with type 2 diabetes mellitus. The results are expressed as the mean ± SD for levels of blood MDA (panel A), arginine (panel B), or blood nitrites (panel C) in RBC samples from control healthy volunteers (n = 90) and in patients with type 2 diabetes mellitus (n = 90). Symbols indicating each experimental group at the top of the panels. Statistics: *p<0.01 against control values (healthy subjects).

**Figure 2:**
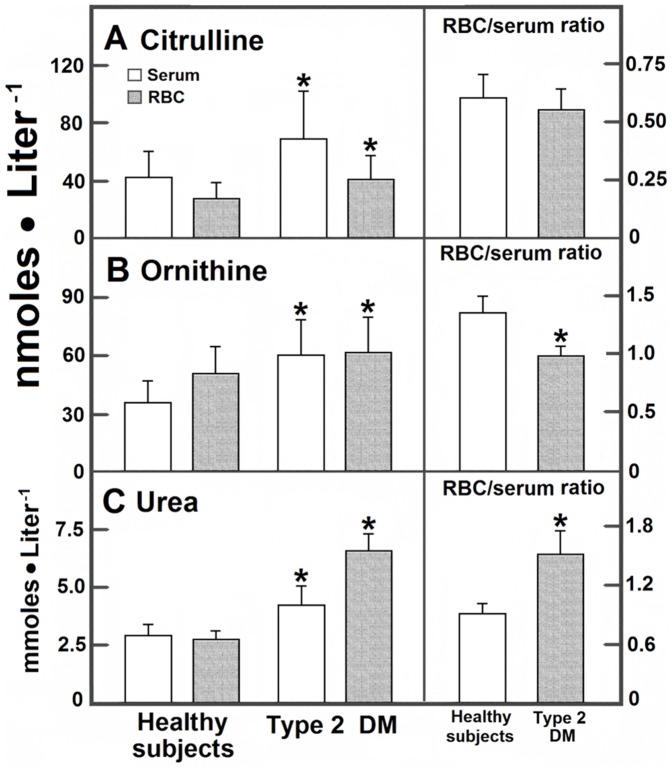
Serum and RBC levels of citrulline, ornithine, and urea from blood obtained from control subjects and patients with type 2 diabetes mellitus. The results are expressed as the mean ± SD for levels of blood citrulline (panel A), ornithine (panel B), or blood urea (panel C) in RBC samples from control healthy volunteers (n = 90) and in patients with type 2 diabetes mellitus (n = 90). Symbols indicating each experimental group at the top of the panels. Statistics as indicated in Fig. 1.

RBC metabolites concentration differed from those found in serum (Figs. 1 and 2), suggesting that RBC could accumulate an important fraction of these metabolites. In serum and RBC from patients with DM, MDA levels (determined as TBARS) were significantly increased in RBC, hence predominating in these cells (Fig. 1); in these patients, the level of serum arginine was not significantly diminished (Fig. 1). In contrast, RBC-arginine was drastically reduced leading to a lower RCB/serum ratio (Fig. 1). In contrast, serum and RBC levels of nitrites were enhanced in DM patients, predominating in RBC when compared with control subjects (Fig. 1). Products of arginine catabolism, namely citrulline and ornithine, were also different in blood samples from DM patients. In controls, blood citrulline largely predominated in serum with an RBC/serum ratio of 0.63±0.10. We did not find a statistical difference in this ratio for citrulline in patients with type 2 DM, but blood citrulline significantly increased in these patients, being the serum levels for this amino acid the most affected by DM (Fig. 2); ornithine showed the opposite, since it was more abundant in RBC. In controls, urea was similarly distributed in both blood compartments, with an RBC/serum ratio of 0.95 (Fig. 2); this by-product was increased in patients with type 2 DM, clearly predominating in RBC (Fig. 2).

### Ratios among Metabolites Participating in NO Production and Arginine Catabolism

Control arginine/nitrites ratio was of 12.8±4.9, 7.9±3.0, and 13.3±4.8, in whole blood, serum, and RBC, respectively, indicating that a substantial amount of arginine remained in RBC, whereas nitrites seemed to rapidly leave these cells (Table 2). In patients with type 2 DM, this ratio was drastically decreased, due mainly to decreased blood arginine level and augmented nitrites production (Table 2). In this context, the arginine/citrulline ratio was of 3.7±1.1 in whole blood from control individuals, mainly attributed to their concentrations in RBC (Table 2). This ratio was significantly decreased in both blood compartments in patients with type 2 DM. Moreover, the arginine/ornithine ratio (3.0±0.8 in control whole blood) was practically the same in both serum and RBC from the control subjects (Table 2). Similarly, DM diminished this ratio in both blood compartments (Table 2). However, the citrulline/ornithine ratio in DM patients did not differ significantly from the control values, suggesting that despite individual variations of ornithine and citrulline concentration in the blood level of DM patients, their distribution between blood compartments was not affected (Table 2). Therefore, data suggest that arginine was proportionally decreased while its putative by-products (nitrites, citrulline, and ornithine) augmented in both, serum and RBC, obtained from DM patients.

**Table 2:** Arginine and its metabolite ratios in blood, serum, and RBC from control subjects and patients with type 2 DM.

	Control Subjects (n = 90)		
Ratio	Blood	Serum	*RBC*
Arginine/Nitrites	12.8±4.9	7.9±3.0	13.3±4.8
Arginine/Citrulline	3.7±1.1	2.6±1.0	5.9±1.9
Arginine/Ornithine	3.0±0.8	3.0±1.0	3.1±0.8
Citrulline/Ornithine	0.8±0.2	1.2±0.4	0.5±0.1
	**Diabetic Patients (n = 90)**		
Arginine/Nitrites	3.1±0.9*	4.6±1.5*	1.6±0.5*
Arginine/Citrulline	1.7±0.6*	1.8±0.7*	1.4±0.5*
Arginine/Ornithine	1.5±0.5*	2.0±0.7*	0.9±0.2*
Citrulline/Ornithine	0.9±0.3	1.1±0.4	0.6±0.2

The results are expressed as means ± SD. Statistics: *p<0.01 as compared to healthy controls.

### Correlations among Blood Metabolites in Serum and Blood Cells in Control Subjects and Patients with Type 2 DM

We looked for correlations among the different measured metabolites. A straight and very significant correlation was found between serum glucose and Hb A_1C_ (r = 0.92; p<0.001). In addition, blood TBARS significantly and directly correlated with serum glucose levels, mainly in serum from patients with type 2 DM (Table 3). Except for blood citrulline levels, where an inverse significant correlation with Hb A_1C_ was noted, blood ornithine, nitrites, and arginine inversely correlated with glucose and/or Hb A_1C_, exclusively in patients with type 2 DM (Table 3). An inverse correlation between serum glucose levels and citrulline was found in both groups. Significant inverse correlations were also found between Hb A_1C_ with ornithine and nitrites (Table 3). Moreover, we found a significant correlation between serum levels of glucose with those of cholesterol (r = 0.72, p<0.001); however, serum cholesterol did not significantly correlate with any of the metabolites here tested.

**Table 3:** Pearson’s correlation coefficients (r) matrix for blood metabolites with serum glucose and RBC-glycated hemoglobin.

Group	Control subjects (n = 90)		Diabetic patients (n = 90)	
	Correlations (r)		Correlations (r)	
Whole blood	Glucose	Hb A_1C_	Glucose	Hb A_1C_
TBARS	N.S.	N.S.	0.69**	N.S.
Citrulline	N.S.	−0.46*	0.32*	0.52*
Ornithine	N.S.	0.38*	0.44*	0.59*
Nitrites	N.S.	N.S.	0.33*	0.48*
Arginine	N.S.	N.S.	−0.74**	−0.63**
Urea	N.S.	0.33*	N.S.	0.53**

Statistical significance: *p<0.01;

** p<0.005.

### Incubation of Isolated RBC with Increasing Concentrations of Arginine

**Figure 3:**
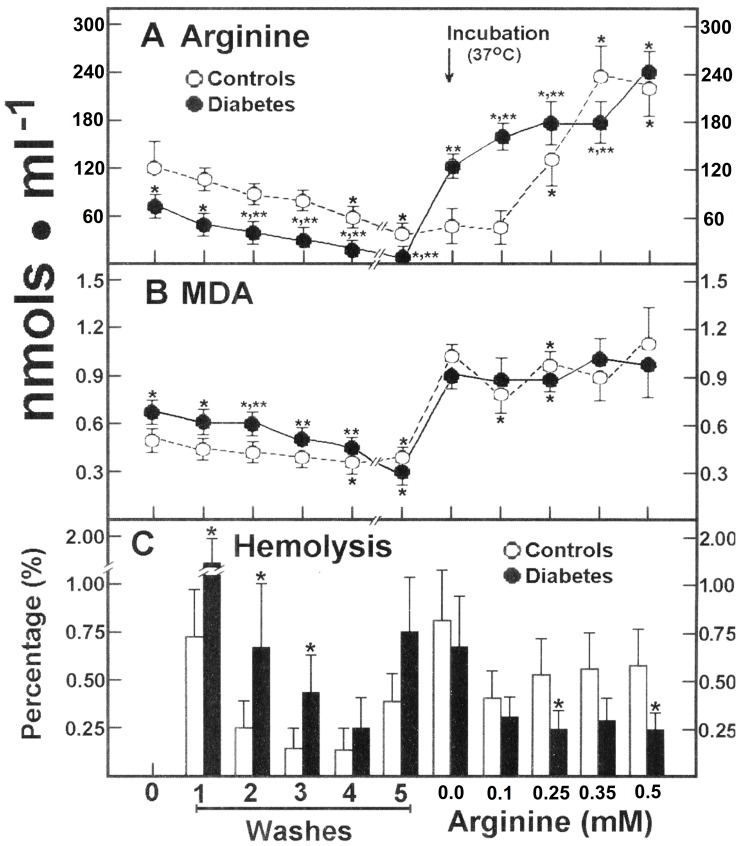
Effect of added arginine on its own release and that of MDA, and rate of RBC hemolysis from control subjects and patients with type 2 diabetes mellitus. The results are expressed as the mean ± SD for levels of released arginine (panel A) and MDA (panel B), in RBC samples from control healthy volunteers (n = 30) and in patients with type 2 diabetes mellitus (n = 30). Panel C shows the rate of hemolysis obtained in each preparation. Start of the incubation at 37°C in the presence of increasing arginine concentrations is indicated by the upper arrow. Symbols indicating each experimental group at the top of the panels. Statistics: *p<0.01 against control basal values (zero) and **p<0.01 vs. the basal metabolite value (zero) in samples from DM patients.

Our data suggested that the serum level of arginine is maintained within normal range in DM patients, through the action of RBC releasing it into the serum. In order to test this, we incubated isolated RBC from healthy controls and DM patients. In RBC from healthy subjects, arginine was gradually released during washing and storage, remaining only 31±7% of the initial level (Fig. 3A). Incubation of these RBC at 37°C did not significantly modify the content of arginine, but after increasing free arginine concentration into the incubation medium, the amino acid was rapidly taken up and RBC concentration linearly increased to saturation in the range of 0.25 to 0.50 mmol/L of added arginine (Fig. 3A). Blood cells from patients with type 2 DM had much lower levels of free arginine, which rapidly escaped into the washing and storage media, remaining less than 6% of the initial value (Fig. 3A). Surprisingly, incubating these RBC at 37°C promoted a drastic increase of arginine in the absence of the added amino acid to the incubation medium; moreover, increasing concentrations of added arginine elicited also a linear uptake of arginine, which was not saturated at the maximum concentrations tested (Fig. 3A). As to the metabolite reflecting oxidant stress (Fig. 3B), washing control RBC and maintaining them at 4°C did not induce an efflux of MDA into the incubation medium, but warming to 37°C readily promoted increased levels of MDA in RBC. When arginine was added into the medium, there was no significant effect on RBC levels of MDA (Fig. 3B). RBC from patients with type 2 DM showed normal basal MDA levels, as previously indicated (Figs. 1 and 3B) and the MDA was indeed washed out reaching similar concentration to that found in control blood cells after storage (Fig. 3B). Incubation of these RBC at 37°C also promoted a drastic increase in MDA, which remained unaffected by incubating with arginine (Fig. 3B). Cumulative hemolysis was significantly higher in RBC from patients with DM than in controls after washing cell packages at 4°C (Fig. 3C). In contrast, there were no differences in hemolysis after incubation at 37°C, but added arginine did confer protection to RBC against hemolysis, being more evident in cells from patients with type 2 DM (Fig. 3C).

### Release of Ornithine and Citrulline from RBC after Incubation with Arginine

**Figure 4:**
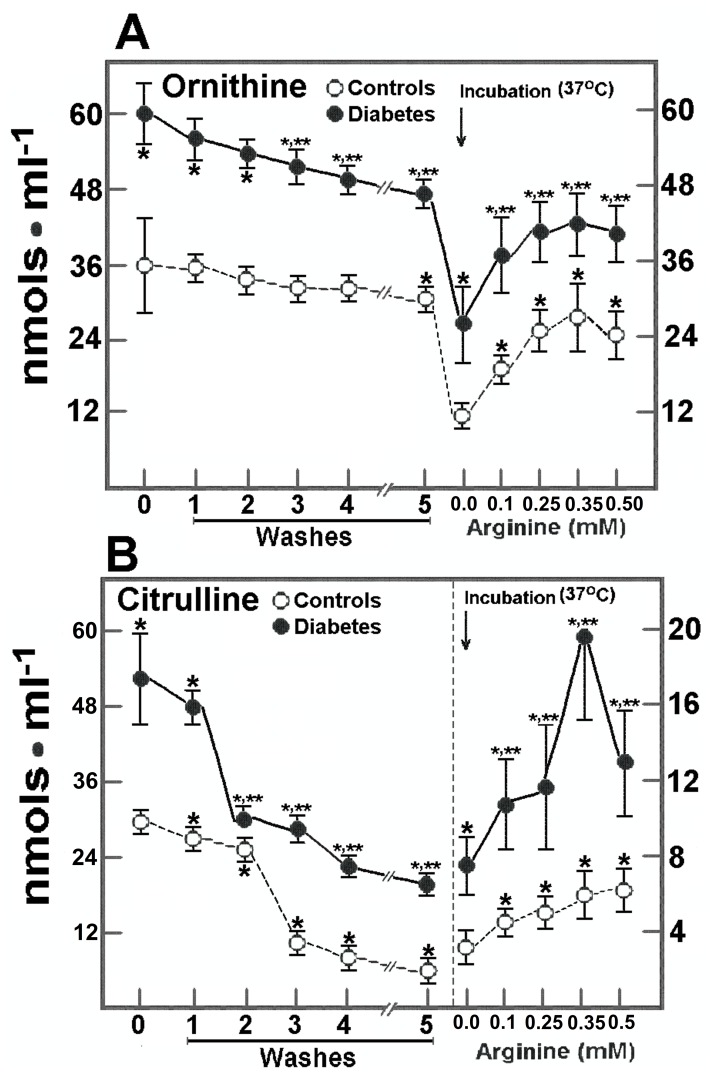
Effect of added arginine on citrulline and ornithine release from RBC from control subjects and patients with type 2 diabetes mellitus. The results are expressed as the mean ± SD for levels of released ornithine (panel A) and of citrulline (panel B) as indicated by symbols at the top of each panel, in RBC samples from control healthy volunteers (n = 30) and in patients with type 2 diabetes mellitus (n = 30). Start of the incubation at 37°C in the presence of increasing arginine concentrations is indicated by the upper arrow. Statistics as in Fig. 3.

The control RBC depicted a slow efflux of ornithine, since only 19% of RBC ornithine left blood cells after washing and storage, but a stronger efflux was noticed after incubation at 37°C (41% of the initial value). Incubating control RBC with arginine induced a gradual elevation in the RBC content of ornithine, which declined when adding 0.5 mmol/L arginine to the incubation medium (Fig. 4A). In the RBC from DM patients, the efflux of ornithine was quite similar to that of control cells after washing, storage, and incubation at 37°C (without arginine), but production of ornithine was noted after adding arginine (Fig. 4A). On the contrary, in control RBC, citrulline was readily released after washing and storage, remaining only a 38±3% of the initial value before incubation. When these RBC were incubated at 37°C, a further decrease of RBC citrulline was found leaving 4% of this amino acid and suggesting that practically all citrulline escaped from the RBC under our experimental conditions (Fig. 4B). After incubation with arginine, RBC citrulline level was linearly enhanced (Fig. 4B). In the RBC obtained from patients with type 2 DM, citrulline was completely released into the medium, remaining only 2.5% of the initial value (without added arginine). In the presence of arginine, RBC-citrulline was enhanced even more in DM patients than in healthy subjects, but started to decrease when these cells were incubated with the highest concentration of arginine (Fig. 4B).

### Release of Nitrites and Urea from RBC after Incubation with Arginine

**Figure 5:**
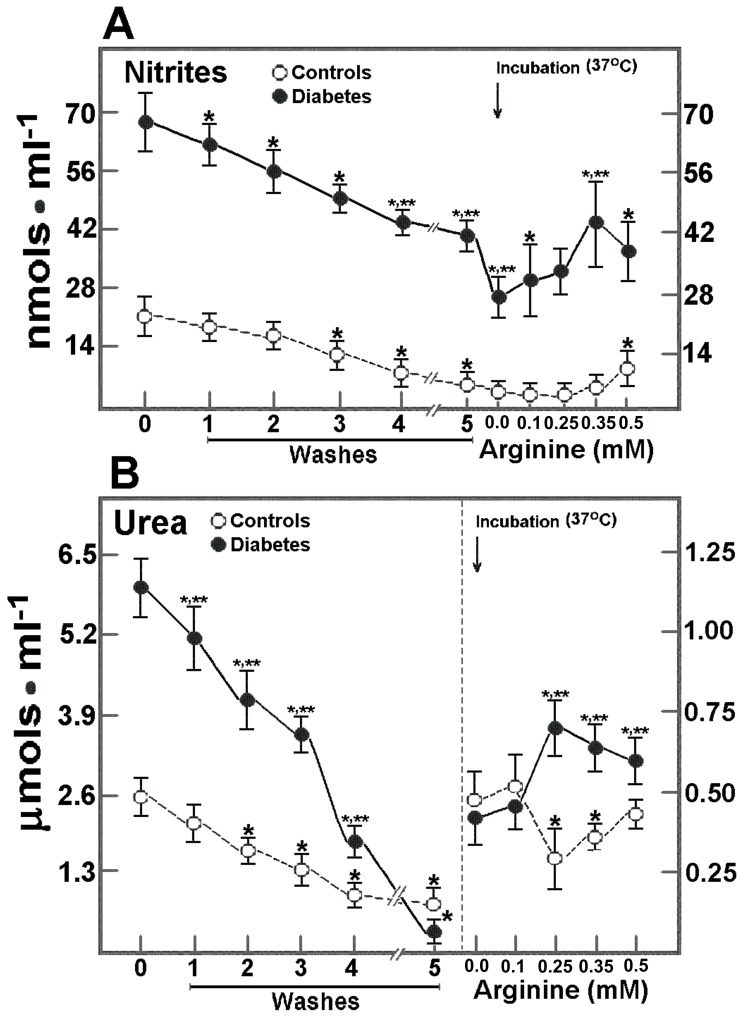
Production and release of nitrites and urea from RBC from control subjects and patients with type 2 diabetes mellitus after incubation with arginine. The results are expressed as the mean ± SD for levels of produced and released nitrites (panel A) and urea (panel B), in RBC samples from control healthy volunteers (n = 30) and in patients with type 2 diabetes mellitus (n = 30). Start of the incubation at 37°C in the presence of increasing arginine concentrations is indicated by the upper arrow. Symbols for each experimental group at the top of the panels. Statistics as in Fig. 3.

The RBC nitrites, which are NO oxidation products, also showed changes after incubating blood cells (Fig. 5A). In controls, efflux of nitrites from RBC was gradual and decreased significantly after washing and storing to a 28±5% of the initial level; incubation at 37°C with arginine did not significantly change RBC nitrites, except at the highest arginine concentration, where a significant 2.8-fold increase was noted when compared with incubating RBC in the absence of arginine (Fig. 5A). Nitrites were quite elevated in RBC from DM patients (Fig. 1) and rapidly decreased after washing and storage, but more RBC nitrites remained in these cells, as compared to control RBC (59±3 vs. 28±5% of the initial level, in controls; p<0.01). However, incubation at 37°C induced a further release of nitrites from RBC of DM patients, whereas the presence of arginine in the incubation medium had no effect on RBC nitrites (Fig. 5A). Since production of ornithine could be linked to the activity of arginase located in RBC, urea, a product of this reaction, was measured under our experimental conditions (Fig. 5B). The urea in control RBC decreased more than 70% after washing and cold-storage; when incubated with arginine (at 37°C) RBC urea was not increased. However, RBC urea in DM patients showed a different pattern (Fig. 5B). These blood cells released more urea after washing and storage, remaining only 13% of the initial value. Moreover, RBC urea was significantly increased, but no linearly, when incubated at 37°C in the presence of increasing arginine concentrations (Fig. 5B).

### Production of ^14^C-ornithine, ^14^C-citrulline, and of ^14^C-urea from ^14^C Arginine by RBC from Healthy Subjects and Patients with Type 2 DM

In order to properly confirm whether the increased amount of RBC ornithine and citrulline measured after arginine incubation is due to enhanced catabolism of this amino acid, identification of radiolabeled products from L-^14^C-arginine was done after separation through thin layer chromatography. Table 4 shows that control RBC incorporated ^14^C-arginine and its concentration in the medium was increased; radio-labeled citrulline and arginine appeared in both, the incubation medium and the pellet of blood cells (Table 4). In control RBC, arginine reached a maximum at the extracellular concentration of 0.35 mmol/L, showing variations thereafter and the same pattern was observed for radio-labeled citrulline; on the other hand, RBC ornithine content was slowly but linearly increased (Table 4). In fact, the decreased amount of citrulline, compared to that of ornithine in control RBC, correlated well with the fact that citrulline is more readily released than ornithine from these RBC into the medium (Table 4). In the case of RBC from patients with type 2 DM, both arginine and citrulline were progressively increased in the incubation medium, but clearly to a lower extent than in control blood cells, whereas ornithine depicted a plateau starting with the presence of 0.35 mmol/L of arginine (Table 4). On the other hand, radio-labeled arginine and citrulline remained higher in RBC, when compared to control cells (Table 4). As to ^14^C-urea, it was clear that control RBC produced urea from arginine in a linear fashion, this was more evident in RBC than in the incubation medium. In addition, RBC from patients with type 2 DM had an increased urea production from arginine in both RBC and incubation medium. It is noteworthy that these RBC incorporated more arginine and produced more citrulline, ornithine, and urea than control cells (Table 4). In fact, from these data, we calculated apparent kinetic constants for NOS and those for arginase; from here, we obtained apparent kinetics for NOS in controls (*Km* = 0.43±0.09 mM, and *Vmax* = 1.06±0.12 nmols/min/mL of RBC), as well as a *Km* = 0.28±0.06 mM and *Vmax* = 1.16±0.16 nmols/min/mL of RBC from DM patients. With L-^14^C-ornithine, we obtained a *Km* of 0.50±0.13 mM and *Vmax* of 0.90±0.14 nmols/min/mL of RBC, for control arginase, compared with a *Km* of 0.23±0.06 mM, and *Vmax* of 0.86±0.15 nmols/min/mL of RBC from diabetic patients. These data would suggest that NOS and arginase affinities for arginine are significantly increased in RBC of patients with type 2 DM, demonstrating a net arginine catabolism by RBC.

**Table 4:** Formation and release of (^14^C)-citrulline, (^14^C)-ornithine and of (^14^C)-urea after incubation with (^14^C)-arginine in RBC from control subjects and patients with type 2 diabetes mellitus.

	Control Subjects (Supernatants)			
Arginine	^14^C-Arginine	^14^C-Citrulline	^14^C-Ornithine	*^14^C-Urea*
0.10 mmols/L	52±4	5±2	3±1	19±3
0.25 mmols/L	114±12	25±3	18±4	20±4
0.35 mmols/L	165±13	65±5	40±5	19±4
0.50 mmols/L	206±16	71±5	50±9	20±4
	**Patients with Type 2 DM (Supernatants)**			
**Arginine**	**^14^C-Arginine**	**^14^C-Citrulline**	**^14^C-Ornithine**	***^14^C-Urea***
0.10 mmols/L	22±3*	8±3	8±2*	14±3*
0.25 mmols/L	43±6*	16±3*	17±4	20±4
0.35 mmols/L	76±11*	27±4*	22±5*	28±5*
0.50 mmols/L	102±14*	34±5*	15±4*	33±6*
	**Control Subjects (RBC)**			
**Arginine**	**^14^C-Arginine**	**^14^C-Citrulline**	**^14^C-Ornithine**	***^14^C-Urea***
0.10 mmols/L	18±4	4±1	4±1	4±1
0.25 mmols/L	56±3	33±4	38±5	5±1
0.35 mmols/L	43±3	15±4	60±5	6±3
0.50 mmols/L	53±3	23±5	70±9	8±4
	**Patients with Type 2 DM (RBC)**			
**Arginine**	**^14^C-Arginine**	**^14^C-Citrulline**	**^14^C-Ornithine**	***^14^C-Urea***
0.10 mmols/L	59±5*	45±11*	65±15*	18±4*
0.25 mmols/L	73±13*	62±12*	84±20*	30±6*
0.35 mmols/L	90±15*	64±12*	85±22*	40±9*
0.50 mmols/L	135±19*	81±13*	60±12	48±9*

The results are expressed as the mean ± SD for levels of produced and released, in nmols per mL, of radio-labeled citrulline, ornithine, and urea, after incubation with (^14^C)-arginine, quantified in supernatants, or in the RBC pellets obtained from control healthy volunteers (n = 30) and in patients with type 2 diabetes mellitus (n = 30). Statistics: *p<0.01 as compared to healthy controls.

## Discussion

Recent findings emphasize the potential key role of amino acid metabolism early in the pathogenesis of diabetes, probably constituting an aid in diabetes risk assessment [32]. However, to our knowledge, this study provides the first evidence that RBC arginine metabolism is altered in patients with type 2 DM, producing increased by-products from arginine catabolism, therefore altering the mechanisms governing this apparent exchange of molecules among organs, blood cells, and serum. Data also confirm that RBC host the enzymatic machinery to metabolize amino acids, such as arginine, besides having efficient transport systems. In this study, DM patients showed increased levels of Hb A_1C_ that correlated with long-lasting hyperglycemia (r = 0.92; p<0.001) and, at a lesser magnitude, with high serum levels of cholesterol, triacylglycerols, and hs-RCP. Endothelial dysfunction can be a major cause of diabetic angiopathy that eventually leads to cardiovascular disease, as a cause of death in diabetes. In fact, improvement of glycemic status and lipid profile are accompanied by amelioration of endothelial biomarkers in subjects with type 2 DM [33].

Increased production of ROS has been attributed to protein glycation [6] and, in turn, increased ROS by-products could result in changes of energy metabolism and antioxidant defense status participating in vascular complications in DM patients [7], [34]. In the DM-associated atherosclerosis, mitochondrial impairment could result from oxidative stress-induced accumulation of advanced glycation end products, with patterns of energy deficiency, which can be reverted by continuous insulin therapy [35]. DM-induced oxidative damage may be more prominent in RBC due to their high content of lipoperoxidative substrates, and a lower rate of ROS scavengers [36]. Here, control blood levels for MDA were similarly distributed in serum and RBC; thus, “free” (serum) MDA could be considered as a “low ROS tone”, similarly to that found in other tissues, whose function is unknown. However, it is also known that an increased amount of LP by-products or of ROS can affect the amino acid and cations transport through RBC membranes, as occurs for cystine transport when human RBC are exposed to oxidative stress [37].

In control subjects, arginine was mainly found in RBC, whereas nitrites were similarly distributed in serum and RBC; in addition, citrulline predominated in serum and ornithine in RBC. In the patients with type 2 DM, both serum and RBC levels of nitrites were enhanced, whereas a drastic diminution of RBC-arginine was also noted (Table 2). These data indicate that DM patients had altered NO metabolism, as previously reported [38]. Amino acids transport in human RBC occurs through three systems, designated L, Ly+, and ASC, and the Ly+ system is highly stereoselective and specific for dibasic amino acids, including arginine [39]. Arginine translocation through RBC membranes is carrier-mediated with simple Michaelis-Menten kinetics, with a high affinity, but with low capacity for transporting the amino acid [40]. Indeed, for some amino acids, erythrocyte transport sometimes exceed that of serum and significant correlation coefficients show that strong serum-erythrocyte relationships exist for arginine and ornithine [41]. Therefore, both serum and RBC are physiologically involved in the blood transport of amino acids in humans.

Based on these considerations, it is clear that RBC have the property of regulating serum levels of some metabolites. Hence, serum arginine levels were maintained within a normal range in detriment of RBC-arginine in DM patients (Table 2). The L-arginine/NO pathway is present in many cells and organs, and the significance of L-arginine could be associated with the biological effects of NO, such as maintenance of normal peripheral vascular resistance and modulation of the vascular wall thromboresistance [42]; increased NO production might be involved in vascular dysfunction and diabetic nephropathy [42]. Additionally, blood levels of citrulline and lysine are diminished, whereas ornithine increases in experimental DM [43]. Therefore, data shown in Table 3 would agree with those reported in the context of chronic hyperglycemia in diabetes, i.e., the effects of advanced glycation end-products on endothelial NO biosynthesis are considerably more important than those of high glucose levels [44]. Moreover, GSH deficiency in RBC is associated with high intracellular concentrations of certain amino acids, particularly ornithine and lysine, and arginine can replace ornithine in low-GSH cells [45]. The aforementioned supports the relations existing among oxidant stress, antioxidant status, transport and metabolism of arginine.

This RBC capacity of buffering serum levels of metabolites is linked to active transport, since the RBC efflux of the different molecules was differential. Indeed, present data showed that, after incubation with arginine, RBC production for ornithine, citrulline, and even urea was increased (Figs. 4 and 5, Table 4), being more evident in RBC from patients with type 2 DM. Hence, RBC might regulate serum levels of amino acids, not only through transport systems but also by an enzymatic machinery capable of metabolizing arginine.

**Figure 6:**
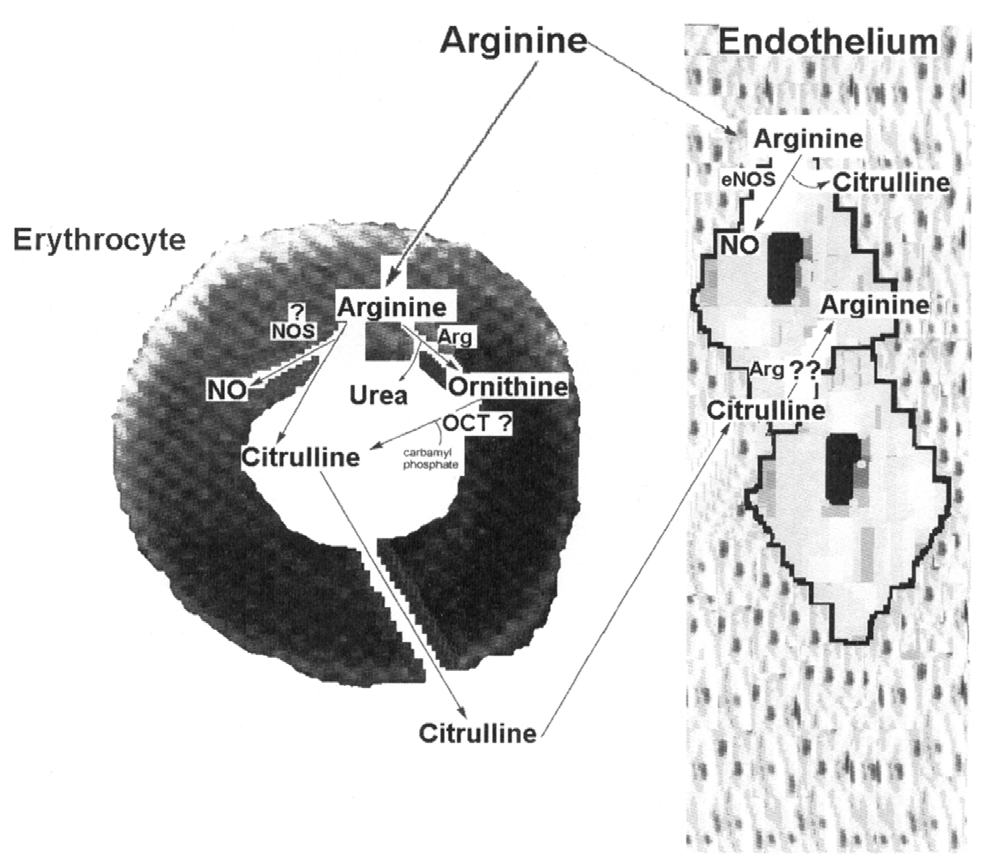
Overview of the possible metabolic pathways for arginine in RBC and their interaction with endothelial cells. Enzymatic routes that can probably directly use or produce arginine, ornithine, urea, or citrulline in the RBC. Key to abbreviations: Arg: arginase; eNOS: endothelial nitric oxide synthase; NO: nitric oxide; NOS: nitric oxide synthase, and OCT: ornithine carbamoyl transferase.

Mature mammalian RBC possess specific, but not concentrative, amino acid transport systems, consisting of a single facilitated-diffusion type of transport mechanism [46]. Hence, RBC contain a large intra-erythrocyte pool of free amino acids actively involved in the inter-organ transport of amino acids [47]. Fervenza et al. [48] demonstrated specific changes in selected RBC membrane transport systems for amino acids during uremia, achieving an altered RBC amino acids pattern, which was not identical to that in serum. Here, it was clear that distribution of metabolites between RBC and serum was not the same and that type 2 DM differentially affected these metabolite patterns. Depletion of L-arginine in endothelial cells is considered barely possible due to high intracellular L-arginine concentrations [49], and the ability of endothelial cells to synthesize L-arginine from L-citrulline [50]. In fact, our data might suggest the existence of an arginine cycle (Fig. 6; scheme), where RBC catabolize a fraction of arginine to citrulline, which, in turn, is taken by endothelial cells to transform it into arginine. The diminished RBC-arginine level and the increased amount of citrulline in both, RBC and serum, compartments, in patients with type 2 DM, also suggest an altered arginine-citrulline cycle between RBC and endothelial cells (Fig. 6).

The production of citrulline from arginine by RBC remains to be explained. It seems to proceed through biosynthesis of NO by NO synthase (Fig. 6), despite that only a very small fraction of exogenous L-arginine is converted via NO into nitrate, and increased conversion of arginine to ornithine occurs without apparent extra-formation of NO [51]. However, there is evidence that RBC from humans have a functional endothelial-type NOS (eNOS), which is localized in the plasma membrane [52] (Fig. 4). Our findings can be also explained by an effective arginase activity, which has been reported to be present in RBC [20]. Blood arginase activity could induce adverse effects by depleting arginine levels, thus suppressing T cells proliferation and favoring metabolic syndrome and endothelial dysfunction [19]. Moreover, arginine depletion promotes a decreased production of polyamines, and these are essential for the proliferation and differentiation of blood cells [53]. Another source of citrulline from arginine-ornithine could be the ornithine carbamoyl transferase activity (OCT) as shown in Fig. 6. However, despite that erythroblasts seem to possess this enzyme, OCT has not been reported in mature RBC. Thus, data agree with the presence of both active enzymes (NOS and arginase) in control RBC, and probably, with an increased affinity for arginine catabolism by RBC obtained from diabetic patients.

The increased urea production by RBC from DM patients might have a negative impact on the functionality of vascular endothelial cells. Urea is rapidly transported across the RBC membrane via a facilitated diffusion pathway, where the RBC urea transporter is encoded by the Kidd locus [54]. From here, it could be expected that blood urea concentration would be similar in both blood compartments, as occurred in blood samples obtained from healthy subjects (Fig. 2). However, in DM patients, besides being drastically increased, urea predominated in RBC with an RBC/serum ratio of 1.59±0.22, which was associated with low intracellular levels for arginine (Fig. 2). These results could suggest that RBC from diabetic patients have altered transport and metabolism functions for these compounds. In this context, elevated levels of arginase and lower serum arginine levels are associated to impaired NO synthesis by endothelial cells [55]. The fact that oral administration of L-citrulline normalizes circulating levels of arginine and total leukocyte counts, improving the wellbeing in patients with sickle cell disease [56], strengthens our main conclusions summarized in Figure 6.

The question arises on how can altered RBC catabolism contribute to the pathogenesis of DM? As mentioned before, arginine decreases serum levels of glucose, homocysteine, fatty acids, and triglycerides, and improves insulin sensitivity in chemically induced diabetic rats [16] and in obese humans with type 2 DM [57]. Moreover, citrulline or arginine supplementation delays the progression of atherosclerosis in obese rabbits [58]. As a whole, these data indicate that arginine plays a role on insulin action; although the mechanism is not known, it is quite possible to be ascribed to NO formation. NO deficiency is a major factor contributing to endothelial dysfunction, which occurs in a variety of metabolic disorders, including diabetes [5]. Deregulation of arginine-produced NO is involved in endothelial dysfunction and endothelium-dependent relaxation, leading to oxidative stress, vascular oxidative damage, enhanced platelet adherence and aggregation, leukocyte adherence, and increased proliferation of vascular smooth muscle cells [5], [17]. Present data indicate that RBC could participate in the equilibrium between arginine metabolism and NO production, and that altered arginine catabolism found in cells from patients with type 2 DM could be involved in endothelial dysfunction, mainly regarding the direct interaction between RBC and endothelial cells.

### Conclusions

The main novelty of the present study relies mainly in demonstrating that RBC are capable of regulating serum levels of molecules possessing metabolic influence through “buffering” their concentrations. Furthermore, RBC can play an additional role in arginine catabolism that is deeply associated with NO production. DM promotes a characteristic pattern of disturbances in the blood levels of the tested metabolites by affecting still unknown properties of RBC. These seem to be linked to metabolite transport systems, putative metabolic pathways, and enzymes, such as arginase, depleting important substrates or enhancing production of molecules potentially harmful, such as urea. These events could be influenced by the rate of oxidant stress. The disturbed capacity of RBC to maintain “normal levels” of serum metabolites could be attributed to chronic exposure of blood cells to high levels of glucose. Moreover, we confirmed that urea can be produced by other cells (RBC) besides hepatocytes or enterocytes in humans, and the pattern of blood metabolites elicited by type 2 DM is probably involved in the specific physiopathology of this disease, i.e., endothelial damage and dysfunction.
